# Cutaneous Leishmaniasis: An Overlooked Etiology of Midfacial Destructive Lesions

**DOI:** 10.1371/journal.pntd.0004426

**Published:** 2016-02-10

**Authors:** Elie Alam, Ossama Abbas, Roger Moukarbel, Ibrahim Khalifeh

**Affiliations:** 1 American University of Beirut Medical Center, Department of Otolaryngology Head and Neck Surgery, Beirut, Lebanon; 2 American University of Beirut Medical Center, Department of Dermatology, Beirut, Lebanon; 3 American University of Beirut Medical Center, Department of Pathology and Laboratory Medicine, Beirut, Lebanon; Hospital Infantil de Mexico Federico Gomez, UNITED STATES

## Abstract

**Background:**

Midline destructive lesions of the face (MDL) have a wide range of etiologies. Cutaneous Leishmaniasis (CL) is rarely reported as a possible cause.

**Methods:**

Fifteen patients with solitary nasal lesions caused by CL were studied. The clinical data, biopsies/scrapings and PCR were collected/performed. Ridley’s Pattern (RP) and Parasitic Index (PI) were documented.

**Results:**

Patients’ age ranged from 1 to 60 years including 7 males and 8 females. The duration of the observed lesions ranged from 1 to 18 months. Clinically, the lesions showed 6 patterns varying from dermal erythematous papulonodular with no epidermal changes to destructive erythematous plaque with massive central hemorrhagic crust. The clinical impression ranged from neoplastic to inflammatory processes. RP varied among the cases [RP 3 (n = 6), RP 4 (n = 3), RP 5 (n = 6)]. All cases show low PI [PI 0 (n = 7), PI 1 (n = 6), PI 2 (n = 1), and PI 3 (n = 1)]. Higher PI was noted in the pediatric group [average age 24 years for PI 0–1 vs. 6.5 years for PI 2–3]. Molecular speciation showed Leishmania tropica (n = 13) and Leishmania major (n = 2). All the patients received Meglumine Antimoniate (Glucantine) injections and had initial cure defined as complete scarring and disappearance of inflammatory signs within 3 months.

**Conclusion:**

Leishmaniasis may cause MDL especially in endemic areas. PCR is instrumental in confirming the diagnosis. MDL caused by CL showed wide spectrum of clinical and microscopic presentation.

## Author Summary

Midline destructive lesions of the face (MDL) involve a process that leads to ulceration and loss of the normal structure. The possible causes vary from infectious to neoplastic. Cutaneous Leishmaniasis (CL) is a neglected tropical disease that is transmitted through the bite of one of the female phlebotomine sandflies and is continuing to spread in endemic and non-endemic regions secondary to environmental and human made changes. CL is rarely reported as a possible cause of MDL. With the influx of over 1,500,000 refugees from Leishmania-endemic areas in Syria seeking shelter from the current conflict in their country, Lebanon, a Leishmania under-endemic country, is facing an epidemic outbreak of CL. In this study, we obtained clinical and laboratory data on 15 patients with solitary nasal lesions caused by CL. We concluded that Leishmaniasis may cause MDL especially in endemic areas and that PCR is instrumental in confirming the diagnosis. Moreover, MDL caused by CL showed wide spectrum of clinical and microscopic presentation.

## Introduction

Midline destructive lesions of the face (MDL) were reported initially in 1897 and over time there have been many terms used to describe them [1]. The most common ones include idiopathic midline destructive disease, lethal midline granuloma, and idiopathic midline granuloma [2]. The various terms used all shared a common descriptive goal which is an ulcerative process leading to cartilage and epithelium loss along with crusting that leads to loss of nasal structure along with a resultant functional and cosmetic deformity.

The differential diagnosis of such lesions is wide and falls in mainly five categories; neoplastic, autoimmune, traumatic, infectious or idiopathic. Each category show some characteristic findings which can suggest it as being thecause, however many of those overlap and this can pose a significant diagnostic dilemma for the treating physician [3]. Despite advancements in the medical field, diagnosis of such lesions is often incorrect or missed. Moreover, the diagnosis of CL is widely overlooked.That's extremely noteworthy knowing that prompt diagnosis is crucial for the proper management and treatment of affected patients [3].

Leishmaniasis is categorized into three forms: visceral, mucocutaneous, and cutaneous leishmaniasis (CL). Leishmania is transmitted through the bite of one of the female phlebotomine sandflies that has been proven to be a vector of Leishmania. Globally, more than 90% of CL cases are reported in Afghanistan, Pakistan, Syria, Saudi Arabia, Algeria, and Iran [4, 5]. However, CL is a neglected tropical disease that is continuing to spread in endemic and non-endemic regions secondary to environmental and human made changes [6, 7]. CL in the Near East is predominantly caused by the Leishmania tropica species. Although not a cause of high mortality, CL can be chronic and disfiguring, producing multiple lesions that induce serious secondary infections and can involve vital sensory organs [5].With the influx of over 1,500,000 refugees from Leishmania-endemic areas in Syria seeking shelter from the current conflict in their country; Lebanon, a Leishmania under-endemic country, is facing an epidemic outbreak of CL (8).In this study, we evaluated cases of CL affecting the midface in 15 subjects.

## Materials and Methods

### Ethics statement

This study was approved by the American University of Beirut Institutional Review Board who waived patient consent since the material deidentified by a third party. Patient data used in this study was anonymized. The material was collected by another research group who approved this research [8].

### Case selection

One hundred and sixty oneSyrian refugees with CL were driven to nearby camp sites and outpatient centers and were examined. Thirty one of these subjects were found to have nasal lesions among other lesions, and 15 of them had nasal lesionsexclusively.Clinical and epidemiological data collected included the lesion’s duration, size, type (wet: exudative or dry: non-exudative), anatomic location, number of lesions, gender, age, and geographic location. Cases were then blindly assessed by a dermatologist (OA) who described the lesions and suggested a differential diagnosis along with the most likely diagnosis. Each lesion was assigned an eruption type (erythematous plaque/patch, papule, papule/nodule, crust, ulcer/verrucous and scar). A clinical impression was proposed to each lesion based on its clinical eruption pattern.

### Microscopic examinations

On the outpatient department premises, a team of trained physicians collected multiple scrapings of the lesions and concurrent punch biopsies. Scrapings were made with the help of a scalpel, pushing in one direction until blood oozes from the inflamed border of the lesion. Multiple drops of the oozing blood were distributed on multiple glass slides. Four millimeter punch biopsies were taken from the border of the lesion of every patient. Skin scrapings were stored at room temperature, whereas biopsies were stored in tightly sealed formalin-filled tubes. A formalin-fixed paraffin-embedded (FFPE) tissue block was prepared from each punch biopsy.Biopsy slides were examined to assess Ridley’s Pattern (RP) and Parasitic Index (PI, Tables 1 and 2) [9].

**Table 1:** Modified Ridley’s Parasitic Index for quantification of Amastigote Load.

Parasitic Index	Number of Amastigotes per standard section
6+	≥100,000
5+	≥10,000
4+	≥1000
3+	≥100
2+	≥10
1+	≥1

**Table 2:** Modified Ridley’s Pattern.

Group	Histopathologic Response
**Group I**	Normal Appearing skin biopsy with patches of collagen degeneration
**Group II**	Predominant severe necrotizing process in the dermis
**Group II**	Dermis involved by a diffuse and heavy mixed inflammatory infiltrate
**Group IV**	Scattered Langhans giant cells and primitive epithelioid histiocytes
**Group V**	Well-Formed granulomas and well-developed epithelioid histiocytes

### Molecular testing

All included cases were sent for PCR confirmation according to a previously published standardized protocol [9]. For FFPE tissue blocks, DNA was extracted from ribbons originating from FFPE. For scraping, the DNA extraction was done using the Pel-Freez (Invitrogen, Carlsbad, CA) kit according to the manufacturer’s instructions. The DNA sample were incubated at 65°C for 30 minutes and then stored at 4°C until analyzed by PCR. The resultant DNA was then quantified using the Biomate spectrophotometer (Thermo, Glasgow, UK).

### Inclusion/exclusion criteria

Only cases that are limited to the midface and had sufficient clinical data and available material for diagnostic confirmation by microscopic examination or PCR were included. All the remaining cases were excluded.

## Results

### Patient demographics

Patients’ age ranged from 1 to 60 years (mean: 22 years) and included 7 males and 8 females. The duration of the observed lesions ranged from 1 to 18 months (mean: 5.5 months). Just, 4 patients recalled an incident of an insect bite.Eight patients migrated from Aleppo, 6 from Homos and 1 from Damascus and all of them resided in different geographic locations within Lebanon. The duration since the patients moved to Lebanon ranged from 1 to 48 months (mean: 8 months).

### Eruption type and clinical impression

**Fig 1:**
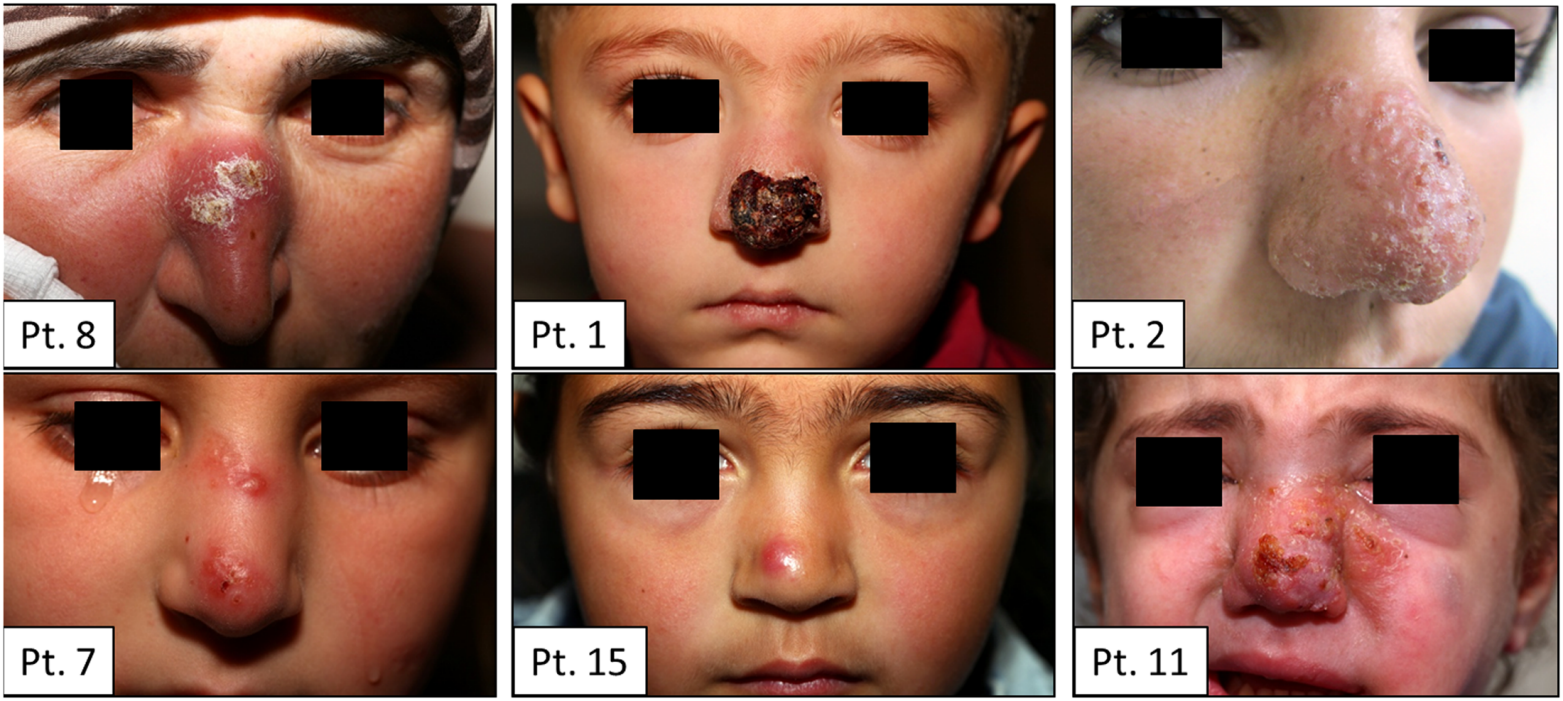
Patients’ images. The most considered differential diagnosis list included Leishmania, Lupus, Lupus Vulgaris, Sarcoidosis whereby seven patients had lesions that fit this differential (see Table 3).

The most frequent pattern of eruption was seen in five patients who had an erythematous plaque with central hemorrhagic crust, followed by 4 patients who had crusted erythematous plaque followed by 3 patients who had a scaly erythematous plaque/patch (see Table 3 and Fig 1). All lesions were dry, but one was wet and found to be Leishmania major.

**Table 3:** Patients’ clinical microscopic and molecular information.

Patient	Gender	Age (years)	Lesion Duration(months)	Dry/Wet	PCR	Ridley's pattern	PI	Molecular species	Eruption pattern	Differential Diagnosis	Favored Diagnosis
**Patient 1**	M	6	9	D	Positive	5	1	T	EPC	Wegener, Lymphoma, Pseudolymphoma, Mycobacterium	Wegener
**Patient 2**	M	12	4	D	Positive	5	0	T	Erythematous Plaque with Multiple papular Component	LSLL	Leishmania
**Patient 3**	M	36	7	D	Positive	4	1	T	EPC	LSLL	Leishmania
**Patient 4**	M	46	8	D	Positive	5	0	T	EPC	LSLL	Leishmania
**Patient 5**	F	12	3.5	D	Positive	3	0	T	SEP	LLS	Lupus
**Patient 6**	M	1	4	D	Positive	3	3	T	CEP	LSLL	Leishmania
**Patient 7**	F	4	2	D	Positive	3	1	T	Papulovesicular and Erythematous lesion	SLPG	Sarcoidosis
**Patient 8**	F	36	4	D	Positive	4	1	M	CEP	LSLL	Leishmania
**Patient 9**	M	12	4.5	W	Positive	3	2	M	EPC	LSLL	Leishmania
**Patient 10**	F	60	18	D	Positive	5	0	T	CEP	LLS	Lupus
**Patient 11**	F	3	3	D	Positive	5	1	T	SEP	LLS	Lupus
**Patient 12**	F	6	1	D	Positive	4	1	T	EPC	Lymphoma, Pseudolymphoma, Sarcoidosis, Leishmania	Leishmania
**Patient 13**	M	34	11	D	Positive	5	0	T	CEP	LSLL	Leishmania
**Patient 14**	F	55	3	D	Positive	3	0	T	SEP	LLS	Lupus
**Patient 15**	F	5	1.5	D	Positive	3	0	T	Erythematous Papulonodular with no Epidermal Changes	SLPG	Sarcoidosis

Gender: M: Male, F: Female

D:Dry, W:Wet

Molecular Subtype: T: Tropica, M: Major

Eruption Pattern:

EPC: Erythematous Plaque with Central Hemorrhagic Crust

SEP: Scaly Erythematous Plaque/Patch

CEP: Crusted Erythematous Plaque

Differential Diagnosis:

LSLL: Leishmania, Lupus, Lupus Vulgaris, Sarcoidosis

LLS: Lupus, Lupus Vulgaris, Sarcoidosis

SLPG: Sarcoidosis, Lymphoma, Pseudolympoma, Lupus

### Microscopic and molecular analysis

**Fig 2:**
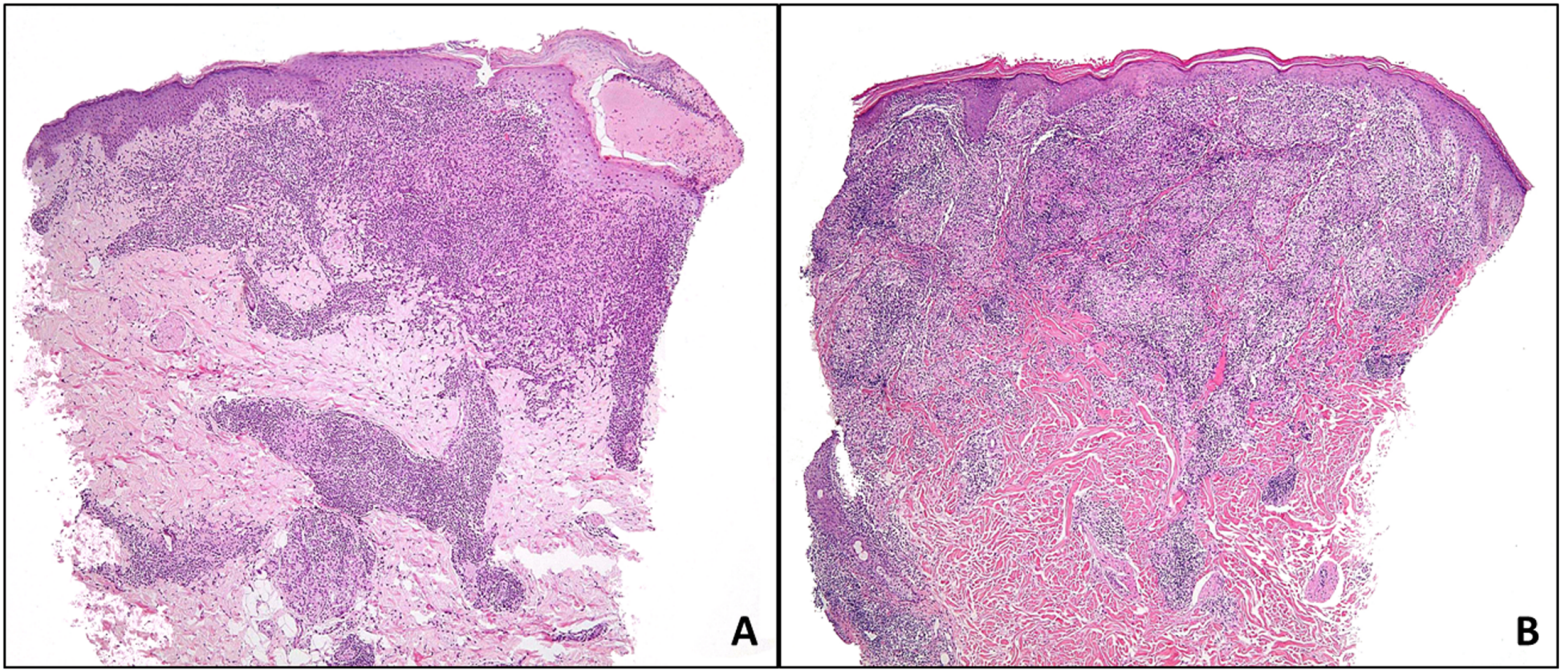
**Case with Ridley’s Patter 3 showing diffuse lymphoplasmacytic inflammatory infiltrates** (A). Case with Ridley’s Pattern 5 showing well-formed granulomas (B).

Microscopically, RP in Our patients ranged from RP 3 (n = 6), RP 4 (n = 3), RP 5 (n = 6, Fig 2). No amastigotes were identified in 7 patients (PI = 0) and the rest had a low PI: PI 1 (n = 6), PI 2 (n = 1), and PI 3 (n = 1). All cases were confirmed by PCR performed on biopsies. PCR performed on scrapings showed same results except for one patient where PCR failed due to bad quality of extracted DNA. Molecular subtype showed 13 patients to have Leishmania tropica whereas two patients had Leishmania major.

### Treatment

Fourteen patients received Meglumine Antimoniate (Glucantime) intramuscularly, and one patient received intralesional injections. All patients had initial cure defined as complete scarring and disappearance of inflammatory signs within 3 months [10]. Group I represents an almost nor-mal appearing skin biopsy with patches of collagen degeneration. Group II shows a predominant severe necrotizing process in the dermis. In Group III, the dermis is involved by a diffuse and heavy mixed inflammatory infiltrate. Group IV shows scattered Langhans giant cells and primitive epithelioidhisti-ocytes. Well-formed granulomas and well-developed epithelioid histiocytes are prominent in Group V

## Discussion

The primary sign ofCL (Old World) is usually a small erythema that develops after a variable period of time at the site where an infected sandfly has bitten the host. The usual progression of the disease is erythema thatadvances into a papule, then a nodule that gradually ulcerates over a period of 2 weeks to 6 months to become thecharacteristic lesion of CL [11]. This usual course is self-limited within one year and is termed “annual sore”.Mucocutaneous leishmaniasis (New World) is characterized by the ability of the parasite to metastasise to mucous tissues by lymphatic or haematogenous dissemination. It is characterized by nasal inflammation and stuffiness followed by ulceration of the nasal mucosa and perforation of the septum [12].

Leishmaniasis has been rarely described to be part of the differential of MDLs. The similarity in clinical symptoms and physical findings makes the accurate diagnosis of MDLs difficult to make. Despite diagnostic improvements and medical advancements, the etiology occasionally remains unidentified [3]. The most common causes of MDLs are nasal extranodal natural killer or T-cell lymphoma (NKTL), Wegener granulomatosis, and cocaine-induced midline destructive lesion. Other causes of MDLs exist but are less frequent and can be grouped into neoplastic, autoimmune, infectious, and idiopathic causes [3, 13, 14].

Crovetto-Martinez et al. described in a recent case report two patients suffering from mucocutaneous leishmaniasis whose presentation and evolution is similar to that of MDLs. The characteristic symptoms of both patients were rhinorrhea, bleeding, and crusting over the necrotic areas.Both cases were misdiagnosed and treated as Wegener's Granulomatosis and NKTL. Moreover, due to therapeutic failure and disease progression, more extensive diagnostic measures were taken to properly diagnose and treat the lesions [15].

Parker et al. discussed the dilemma and difficulty of diagnosing midline lesions. The authors stressed on the importance of accurate diagnosis starting with the history and physical findings and how these findings can be attributed to a possible cause. In addition, they concluded that it is imperative to take multiple tissue specimens from possible sites for proper diagnosis and treatment [3].

In this study, we described 15 cases of Syrian Refugees in Lebanon presenting as facial midline lesions. Due to low PI in all the cases, the diagnosis of Leishmaniasis could have been overlooked; however the index of suspicion was high due to a CL breakout among the refugees. This in turn led to further confirmation via PCR.

As described in a previous study by Saab et al, the manifestations of CL are broad and may resemble not only inflammatory but also neoplastic diseases [16]. Similarly, in MDLs there is a variation in microscopic and clinical presentation. This is where PCR is instrumental even in cases where PI is low, especially when dealing with an endemic population, and knowing that low PI is a common presentation microscopically [9].

All patients in this study had a low PI (3 or less).When grouped into two sets, PI 0–1 and PI 2–3, to determine any difference in lesion duration and patient age between the 2 groups. The difference in lesion duration was not significant [6 months (PI 0–1) vs. 4 months (PI2-3)]. However, the difference in patient age was significant [average age was 24 years for PI 0–1 versus 6.5 yearsfor PI 2–3], whereby the pediatric group had a higher PI which represents the majority of our patients (9/15). Cutaneous lesions involving L. major typically manifest after a short incubation period of 1 week to 2 months with an exudative or ‘wet ulcer’. This study revealed that, in contrast to common thoughts that Leishmania major is characterized by wet lesions; one of our patients had a dry lesion and was found to be Leishmania major.

The most adopted treatment modalities of CL are Meglumine Antimonate (Glucantime) and Sodium Stibogluconate (Pentostam). Both are on the WHO essential drug list for CL. In our study we used the traditional treatment, Glucantime, because it is more readily available and less expensive than Pentostam [10].

In summary, this is to our knowledge the largest case series in the English literature describing CL as a cause of midline facial lesions. We were able to highlight the importance of having a high index of suspicion for Leishmaniasis and including it in the diagnosis of midline facial lesions especially in endemic areas. This holds true even in patients with a low PI whereby PCR confirmation becomes instrumental. This study further stresses on the variation in patients with midline facial CL clinically and microscopically, and also highlights the impact of human migration on CL evolution in non-endemic areas.
